# Uncovering Missing Heritability in Rare Diseases

**DOI:** 10.3390/genes10040275

**Published:** 2019-04-04

**Authors:** Tatiana Maroilley, Maja Tarailo-Graovac

**Affiliations:** 1 Departments of Biochemistry, Molecular Biology and Medical Genetics, Cumming School of Medicine, University of Calgary, Calgary, AB T2N 4N1, Canada; tatiana.maroilley@ucalgary.ca; 2 Alberta Children’s Hospital Research Institute, University of Calgary, Calgary, AB T2N 4N1, Canada

**Keywords:** missing heritability, rare disease, genome sequencing, long/short read sequencing, bioinformatics, variant detection, variant annotation, variation databases

## Abstract

The problem of ‘missing heritability’ affects both common and rare diseases hindering: discovery, diagnosis, and patient care. The ‘missing heritability’ concept has been mainly associated with common and complex diseases where promising modern technological advances, like genome-wide association studies (GWAS), were unable to uncover the complete genetic mechanism of the disease/trait. Although rare diseases (RDs) have low prevalence individually, collectively they are common. Furthermore, multi-level genetic and phenotypic complexity when combined with the individual rarity of these conditions poses an important challenge in the quest to identify causative genetic changes in RD patients. In recent years, high throughput sequencing has accelerated discovery and diagnosis in RDs. However, despite the several-fold increase (from ~10% using traditional to ~40% using genome-wide genetic testing) in finding genetic causes of these diseases in RD patients, as is the case in common diseases—the majority of RDs are also facing the ‘missing heritability’ problem. This review outlines the key role of high throughput sequencing in uncovering genetics behind RDs, with a particular focus on genome sequencing. We review current advances and challenges of sequencing technologies, bioinformatics approaches, and resources.

## 1. Introduction

Heritability is a measure that estimates the proportion of a phenotypic trait variability that is genetic in origin (i.e., could not be explained by the environment or random chance). The ‘missing heritability’ problem term was first coined by Brendan Maher in 2008 [1], mainly to describe unmet expectations from the human genome project combined with promising modern technological advances, such as genome-wide association studies (GWAS), to uncover genetic components of common traits and diseases [1]. Although the problem of ‘missing heritability’ has been mostly (read exclusively) associated with common and complex diseases in the medical research field [1,2], rare diseases also face ‘missing heritability’ problem despite the state-of-the-field technological advances [3].

Rare diseases (RDs) are mostly genetic diseases that are defined as life-threatening or chronically debilitating disorders affecting a small number of people (fewer than 5 per 10,000) [4]. Some 7000 RDs have been reported to date (see ORPHANET [5] and OMIM for Online Mendelian Inheritance in Man [6] databases) and new syndromes continue to be described, making the RDs quite common overall. An estimated 350 million people in the world suffer from a rare disease and approximately 50% of those are children. In Canada, this represents approximately 1 in 12 people according to the Canadian Organization for Rare Diseases (CORD).

Traditionally, clinical genetic tests for diagnosing RD patients have involved high resolution molecular single-gene tests (e.g., Sanger sequencing), low resolution genome-wide cytogenetic tests (e.g., G-banded karyotype) or microarrays have achieved a diagnostic success rate of ~10% [3]. While the GWAS had uncovered new associations in common diseases, this approach was not adaptable to RDs, due to genetic and phenotypic heterogeneity combined with the rarity of individual conditions, and the unavailability of large cohorts. It is only the crucial technological advances in high throughput sequencing (HTS) and the bioinformatics field that have enabled unprecedented opportunity to accelerate diagnosis and discovery in RDs [3,7,8,9]. However, even after almost a decade of HTS applications in RD patients, the majority of RD patients remain without genetic answers [3].

Here, we focus on the concept of the ‘missing heritability’ problem in the rare disease research field. We review the HTS approaches used so far, and highlight the potential of genome sequencing to uncover ‘missing heritability’ in RDs, with particular attention to types of sequencing technologies, bioinformatics approaches used, and available resources on ‘normal’ variation within populations. We conclude with future perspectives.

## 2. Complexity of Rare Diseases

### 2.1. Heterogeneity

phenotypic heterogeneity refers to strikingly different phenotypes associated with different variants of the same gene. For example, variants in *TRPV4* have been reported in more than 10 different dominant disorders, from various forms of skeletal disorders (e.g., Brachyolmia type 3, Parastremmatic dwarfism), to neuromuscular disorders (e.g., Hereditary motor and sensory neuropathy, type IIc, various forms of Spinal muscular atrophy) [6,10]. Similarly, variants in *FLNA* have been reported in various X-linked dominant (XLD) and recessive disorders (XLR), such as Periventricular Heterotopia 1, various malformation syndromes (e.g., XLD Otopalatodigital syndrome, XLR Frontometaphyseal dysplasia) and others [6]. Recently, we [11] and others [12] have associated heterozygous variants in the *ATP1A1* to human diseases, either an inherited dominant Charcot-Marie-Tooth type 2 disease [12] or a more severe condition due to de novo variants with major features of renal hypomagnesemia, refractory seizures, and intellectual disability [11]. Another example of an emerging rare disease with phenotypic heterogeneity is Glutaminase deficiency. While a homozygous copy number variant (duplication) in *GLS* was associated with autosomal recessive spastic ataxia and optic atrophy in two brothers from a consanguineous family [13], homozygote loss of functional variants (e.g., nonsense and frameshift) were associated with severe neonatal Epileptic encephalopathy and death before 40 days [14]. Thus, with the discoveries of new genes related to human diseases (like *ATP1A1* and *GLS*), it is clear that phenotypic heterogeneity continues to play an important role, and must be considered when interpreting the data.

Genetic Heterogeneity, on the other hand, is defined as variations in distinct genes (two or more) that produce the same or similar phenotypes, either biochemical or clinical. Beyond the phenotypic heterogeneity, the genetic heterogeneity of RDs poses substantial diagnostic challenge. The degree of heterogeneity varies between different diseases. For example, thus far cystic fibrosis had only been associated with variants in *CFTR* [6], while tuberous sclerosis had only been associated with *TSC1* and *TSC2* [15]. These are good examples of currently no known (cystic fibrosis) or low (tuberous sclerosis) genetic heterogeneity. On the other hand, retinitis pigmentosa is an inherited degenerative disease resulting in severe retinal dystrophy and visual impairment mainly with onset in infancy or adolescence. It is usually diagnosed by a clinical exam and electrophysiological recordings, but a genetic diagnosis requires a multi-gene approach since more than 60 different genes had been associated with monogenic retinal disorders [16]. While retinitis pigmentosa may be considered to be an example of moderate heterogeneity, intellectual disability with more than 800 different gene associations [17] exemplifies substantial heterogeneity in human genetic diseases. Thus, considering phenotypic/genotypic heterogeneities in RDs is crucial for a successful approach to diagnosis.

### 2.2. Mutation Spectrum

**Figure 1:**
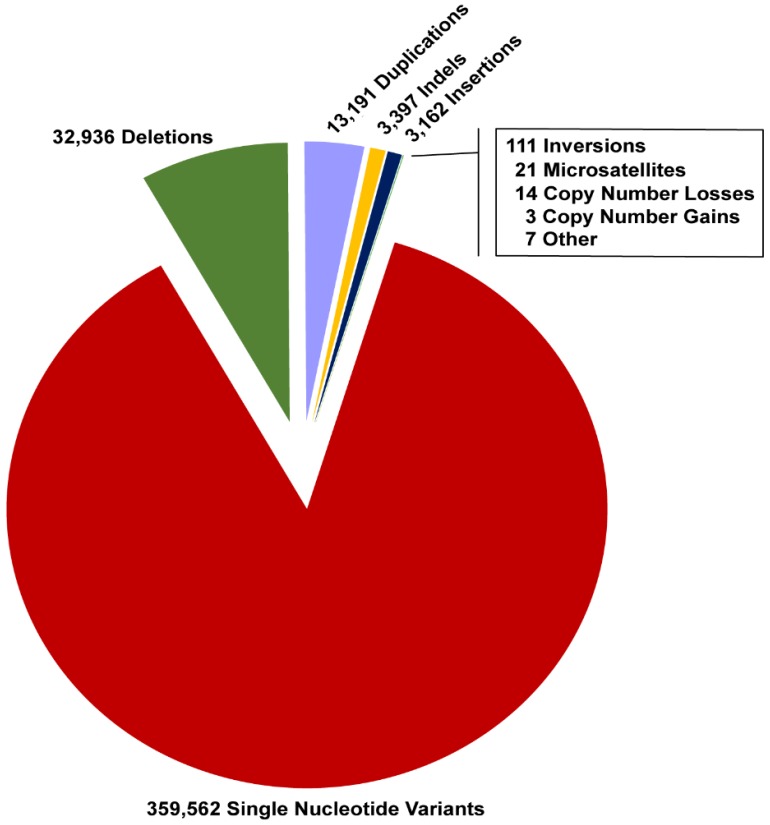
ClinVar variome. Representation of ClinVar variant types (as of December 2018). About 13% were structural variants. The annotation of variants is according to sequence ontology [19].

ClinVar [18], a freely accessible repository of human variation, summarizes reports of variants related to human phenotypes with an evaluation of pathogenicity (likely/benign, uncertain significance, likely/pathogenic) and the potential source of supporting evidence. As of December 2018, more than 412,000 variants were available in ClinVar. Importantly, of those 13% (*n* = 52,424) were variants other than single nucleotide variants (SNVs) (Figure 1).

**Figure 2:**
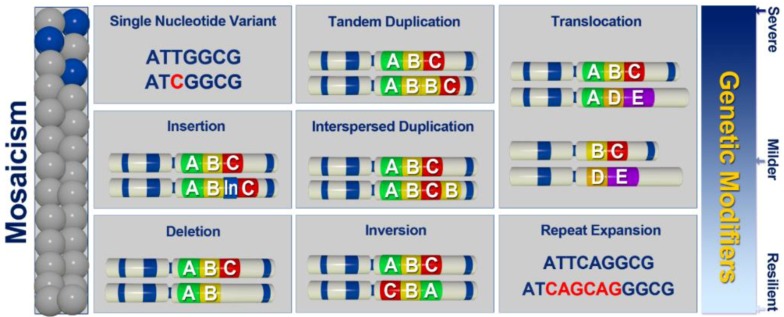
Uncovering missing heritability. A spectrum of variants, beyond the SNVs (single nucleotide variants), contributes to human genetic conditions as either germline or somatic variations. In addition, different types of variants, such as large insertions (including mobile element insertions (MEI)), deletions, duplications, as well as translocations, inversions, repeat expansions and other complex changes may be the source of genetic modifiers with the capacity to alleviate or exacerbate the effect of the primary pathogenic variant, and thus contribute to phenotypic variability (severe-mild-none).

Most of the well-described monogenic diseases display a spectrum of gene-inactivation mechanisms [15,18,20,21]. For example, in patients with a clinical diagnosis of tuberous sclerosis, a spectrum of heterozygous variants affecting *TSC1* and *TSC2* had been described [15,20]. The variants range from SNVs resulting in missense, nonsense, splice-site changes, to structural variants (SVs), such as large deletions and duplications [20]. Interestingly, somatic, rather than germline variants, (in *TSC1* and *TSC2*) were identified in patients resistant to conventional diagnostic approaches [15,20]. Furthermore, in recent years HTS technologies revealed another type of SV termed chromothripsis, a type of chromosomal rearrangement with massive and complex clustered SVs that leave the affected genomic region changed beyond recognition [22]. Although chromothripsis had been predominantly associated with somatic genome instability (e.g., cancer), it had also been reported in individuals with severe congenital abnormalities [23] as well as in the striking case of spontaneous recovery in a patient with WHIM syndrome [24]. Given the variety of genetic mechanisms in gene inactivation, a holistic approach to assessment of individual genomes, including large insertions (such as mobile element insertions (MEI)), deletions, duplications, as well as translocations, inversions, repeat expansions, and other complex changes (Figure 2) would be a desired approach to discovery of functional variants in patients with rare disease.

### 2.3. Phenotypic Variability

Multilocus Genetic Inheritance contributes to phenotypic variability and subsequent diagnostic difficulty in patients with RDs. With the advent of HTS, it had been recognized that phenotypic variability or atypical presentation of a disease may be due to two or more genetic conditions with overlapping (blended) or discrete (composite) manifestations [25,26,27]. Newly discovered genetic conditions may also co-occur with another genetic condition(s) [28,29] (e.g., *NPL* and *GJB2* composite effects in a patient with sialuria, exercise intolerance/muscle wasting, cardiac symptoms, and deafness) [28]. Thus, considering multiple diagnoses in a patient is important in presumed monogenic disorders, especially the ones with atypical ‘ultra’ rare phenotypes [30] and/or substantial phenotypic variability [31] before a conclusion on expanded clinical presentation of a monogenic disease is made.

Beyond composite and/or blended effects of two or more genetic conditions, an increasing number of RDs is being reported where mutations in two or more genes need to co-occur for the disease to manifest. DIDA [32], a database on digenic diseases compiles information on 44 different digenic diseases and 213 of their corresponding digenic combinations [33]. For example, in ciliopathies, digenic compound heterozygous inheritance is repeatedly reported (e.g., Joubert syndrome; one heterozygous variant in *CEP41* and another in *KIF7*) [34]. Importantly, recent findings suggest that oligogenic inheritance may explain missing heritability problem in multiple genetic diseases classically considered to be monogenic, such as Long QT [35] syndrome, Holoprosencephaly [36] and others [33,35].

Genetic Modifiers are important contributors to phenotypic variability (Figure 2). As modulators, these variants may alleviate or exacerbate the effect of the primary pathogenic variant leading to variable penetrance and expressivity of RDs and poor genotype-phenotype correlations even among the siblings. The extent of variation of any individual genome, combined with a known/expected property of genetic modifiers (variants of modest effects, not necessarily rare, also likely to affect non-coding regions) makes it difficult to identify these in small patient cohorts, typical for RDs. However, large-scale sequencing projects that combine phenotypic information are proving to be invaluable resources for assessing penetrance and expressivity in RDs [37,38], and thus the potential effect of genetic modifiers [39,40].

### 2.4. Unknowns

Unknown Gene-Disease Associations contribute to missing heritability in RDs. OMIM (Online Mendelian Inheritance in Man) database [6], daily updated, makes the inventory of the described and published disease-related phenotypes with the causing genes and variants. To date, OMIM contains information on more than 15,000 genes and more than 8000 human disease phenotypes with a suspected Mendelian basis [6]. However, for more than 3000 phenotypes there is no known molecular basis of the disease. Given the rate at which new gene-disease associations are established [41], it is expected that the next decade will establish the majority of the currently unknown gene-disease associations, and thus facilitate better diagnostic success in patients with RDs.

Unknown Genetic Mechanisms continue to be an important possible cause of missing heritability in RDs. For example, non-coding genome (~98% of the human genome) remains largely unexplored, yet emerging studies reinforce the importance of considering these variants in RD patients [42]. Similarly, recently described promoter epimutation [43] or allelic imbalance due to untranslated (UTR) variations [44] are some examples of not routinely screened genetic mechanisms that may cause unexplained RDs.

## 3. High Throughput Sequencing—Untangling Complexity

### 3.1. Exome Sequencing

Over the last decade, HTS has had a substantial impact on RDs by improving the likelihood of reaching a diagnosis. In particular, exome sequencing has emerged as an endorsed approach, mainly due to its cost-effectiveness and practicality.

Gene Panel Sequencing refers to a type of HTS approach where a subset of known disease regions or known disease genes is targeted for sequencing. Gene panels can be of various sizes, from only two genes to thousands of genes, with the most comprehensive panels targeting all exons of the genes currently known to be associated with monogenic disease (e.g., Illumina’s TruSight One ~4800 genes or TruSight One Expanded ~6700 genes). Panels offer the advantage of limiting the search for pathogenic variants to known disease gene set [45,46]; thus, circumventing the need for time-consuming interpretation of potentially unrelated variants and/or incidental findings (IFs). However, gene panels may result in missed or incomplete diagnoses, due to limited ability to address: (1) heterogeneity, (2) variability due to multiple diagnoses where one or more conditions may not be included on the panel, (3) novel genetic diseases and/or (4) genetic mechanisms of the disease due to limited capacity of the panel to detect a spectrum of gene-inactivation mechanisms.

Whole Exome Sequencing (WES), on the other hand, simultaneously targets an entire set of protein-coding genes and allows a more comprehensive approach to uncovering missing heritability in RDs. An effective compromise between cost-effectiveness (e.g., targeting exome, a small part of the genome, <2%) and inclusion (e.g., most of the coding gene regions), WES had enabled unprecedented discoveries. These include, but are not limited to, discoveries of novel gene-disease and genotype-phenotype associations [6], unexpected role of somatic mosaicism in undiagnosed cohorts [15,47,48], as well as novel discoveries of causes of phenotypic variability (e.g., multiple genetic diagnoses in a single patient [25,26,27]). Moreover, WES effectively improved the diagnostic success rate well beyond the ~10% diagnostic rate of high resolution molecular single-gene tests (e.g., Sanger sequencing), low resolution genome-wide cytogenetic tests (e.g., G-banded karyotype) or microarrays [3]. While the diagnostic rate of WES varies widely depending on disease type, patient selection and type of the WES test (e.g., singleton-WES analyzing only the proband vs. trio-WES including the proband and two unaffected relatives, in most cases parents) [3], the overall diagnostic rate of trio-WES for RDs is estimated to be between 30% and 50% [3,49,50]. While WES had played a pivotal role in addressing multiple levels of complexity associated with deciphering RDs, it is still a test limited to a very small portion of a genome and exome-capture technologies [51]. This limitation of WES may explain persistent missing heritability in RDs, including the RDs with well-established clinical diagnosis [15,52].

### 3.2. Genome Sequencing

Unlike targeted sequencing approaches, whole genome sequencing (WGS) enables untargeted view of the entire human genome, and thus is the most comprehensive test with the potential to identify every genetic variation that plays a role in human disease, causing either primary or secondary clinical features, or modifying the primary disease-causing variant (Figure 2). However, since sequencing human genomes became affordable, there have been mixed reports on the benefits of genome sequencing as opposed to exome sequencing in RDs. Some report marginal benefit [3,53,54], while others report a substantial benefit [55,56]. Nonetheless, all of these studies demonstrate that WGS facilitates discoveries not possible using exome sequencing (Table 1). For example, we recently reported on a family with a biochemical diagnosis of Dihydropyrimidine Dehydrogenase Deficiency (DPDD) in three members of one family [52]. Thus far, the only known genetic cause of DPDD is the alteration of *DPYD* resulting in autosomal recessive inheritance. While one member of the family received a genetic diagnosis (compound heterozygote for two *DPYD* variants), two family members with a confirmed biochemical DPDD remained only with partial genetic diagnosis despite clinical genetic tests including WES. Indeed, only one heterozygous *DPYD* variant was identified in these individuals, while the second variant expected for this recessive condition was missing [52]. It was only by WGS (Illumina short-read) that we were able to resolve the ‘missing heritability’ problem in this family, which was due to a complex SV, an imperfect >100Kb inversion with breakpoints in introns 8 and 12 and 4 bp deletion in *DPYD* [52]. Recently, a role of short repeat expansions in ‘missing heritability’ was demonstrated by identifying a cause of Benign Adult Familial Myoclonic Epilepsy (BAFME) [57]. Using single-molecule, real-time sequencing of BAC clones and Nanopore sequencing of genomic DNA, Ishiura et al. (2018) identified the same abnormal expansions of TTTCA and TTTTA repeats in introns of several different genes (*SAMD12*, *TNRC6A* and *RAPGEF2*), suggesting that it is the repeat expansion that is the cause of pathogenesis in BAFME rather than one of these genes specifically [57]. These and other examples (Table 1) clearly show the potential of WGS to uncover missing heritability, in particular variants other than SNVs, as well as variants located in a region not captured by WES, such as deep intronic variants (Table 1). In fact, Brendan Maher, who broached the concept of missing heritability over a decade ago, had already suggested that perhaps it makes sense to stop relying on SNV-gnostic technologies (e.g., GWAS in common disease and exome sequencing in RDs), and start looking for other types of variation as structural variants (SVs) via genome sequencing [1]. Although it is clear that WGS surpasses exome sequencing in its ability to uncover more (Table 1), the question remains whether it is possible to enhance the discovery and diagnostic potential of WGS beyond the currently reported rates [3,55].

Short-read sequencing is a type of HTS also known as second- or next-generation sequencing that could be further sub-divided into two categories: (1) sequencing by ligation (e.g., Complete Genomics and SOLiD platforms) and (2) sequencing by synthesis (proposed by Illumina, Qiagen, 454 pyrosequencing and IonTorrent platforms). These sequencing approaches allow high-throughput analyses with low error rate (Illumina accuracy rate >99.5%) and affordable per base costs. However, the short reads (typically 100 to 400 bp in length [8]) are challenging for accurate mapping (e.g., resolution of pseudogenes) and the detection of SVs [58].

Long-read sequencing is a type of HTS known as third-generation sequencing that also could be sub-divided into two main categories: (1) single-molecule real-time sequencing approaches (SMRT, e.g., Pacific BioSciences, PacBio [59] and MinION, PromethION from Oxford Nanopore Technologies [60,61,62] and (2) synthetic long-read approaches proposed by Illumina and 10X Genomics. 

The Nanopore sequencers are able to produce on average 7–8 kb long reads and PacBio 10–15 kb long reads which may facilitate better detection of SVs as a result of more accurate alignments and better likelihood for detection of repetitive regions and tandem repeats [72]. However, there are many limitations associated with long-read sequencing technology, such as (1) significantly lower throughput; (2) higher per-sample sequencing cost (e.g., human WGS at 30Xcoverage is ~30-fold more expensive using PacBio than Illumina); (3) high error rates of >10% [8,73]; and (4) less resources of the available bioinformatics tools.

Holistic/comprehensive Approaches: Despite the advantages and disadvantages of both the short- and long-read sequencing technologies, both of these were successfully utilized to uncover a spectrum of SVs not easily/detectable by other approaches (Table 1). For example, short-read sequencing WGS successfully detected variants, such as deletions, duplications, inversions, repeat expansions, translocations, mobile element insertions, as well as complex structural variants (e.g., duplication-inversion-inversion-deletion or chromothripsis) (Table 1). Similarly, long-read sequencing had been successfully applied to detect SVs (Table 1). A combined approach may also be a possibility, as demonstrated by several studies where combining Nanopore and Illumina technologies (Table 1) helped resolve complex SVs [65,70] or synthetic long-read technology may be considered (10X Genomics/Illumina). This technology re-builds long reads in silico using barcodes in existing short-reads, and thus could potentially bypass issues related to the cost, error rates, and throughput of true long-read sequencers [73]. Nonetheless, we believe that in order to maximize holistic potential of WGS, besides the detection of a variation spectrum (Figure 1 and Figure 2, Table 1), good coverage is desired in order to reliably call variants in both homozygous and heterozygous states, as well as somatic mosaicism, an emerging cause of missing heritability [15,47,48].

Currently, short-read sequencing technology has been very well positioned to lead the way in comprehensive genomics (Table 1), and the emerging computational approaches may effectively address the limitations of short-reads [8] (Table 2). For example, the recently developed ExpansionHunter uses PCR-free WGS short-read data to identify long repeat expansions, addressing the problem of identifying repetitive variation that is longer than the sequencing read itself [74]. Considering that just some 20 tandem repeat diseases have been described to date [75], and the fact that the repeatome (all repetitive or repeat-derived DNA sequences in a genome) represents a substantial source of variation in humans [75,76,77], is suggestive that with tools like ExpansionHunter [74] and GangSTR [78], we are likely to uncover many more causes of missing heritability (both germline [57] and somatic, Figure 2). Beyond the repeatome, other SVs represent a substantial potential for individual variation [79] (estimated to be up to 10-fold larger than that of SNVs) [80], and mobile elements (~45% of the human genome [81]) also play an important role (Table 1) [82]. Many tools had been developed for a specific type of SVs and continue to be tested and evaluated (Table 2). Genome sequencing has already been shown to be at least as sensitive as microarrays in discovery of CNVs, both germline, de novo and somatic [83], using Canvas [84,85] (Table 2), and data mining/machine learning algorithms are being developed to assess performance and to merge calls from various SV-calling algorithms [86,87].

### 3.3. Genome and Phenome Resources

Reference Genome: A crucial step of HTS bioinformatics pipelines is the read mapping with the following scenarios: (1) alignment along a reference genome; (2) alignment along a personalized genome; (3) de novo alignment or (4) alignment-free process. The most widely used approach is the alignment along a reference genome. A human reference genome is an assembly of sequenced DNA from a number of people, which is stored in a database in its digital form. It provides a haploid mosaic of different DNA from each donor, and thus not any single person in particular. For example, the Genome Reference Consortium human genome, build 37 (GRCh37/hg19) released in February 2009, is derived from 13 anonymous volunteers from Buffalo, New York [99], and the new build GRCh38/hg38 (release in December 2013) contains the same DNA but with more than 100 gaps that were present in hg19 now closed in hg38, some using Nanopore sequencing [100]. One disadvantage of the widely used read mapping via the reference genome approach is the assumption that the 13 volunteer genomes are representative of the genetic background of various populations subjected to genome/exome sequencing, which is unlikely to be the case. First, it has been shown that the human reference genome contains only an allele of O blood type of the ABO blood groups [101] and misses segments of DNA present in other populations [102], and additionally, it harbors some 20,000 ultra-low frequency alleles [103]. Thus, alternative approaches, such as ethnically concordant synthetic human reference sequence [104] or genome graphs (a mathematical graph of variation missing from the reference) [105] may play an important role in improving unique read mapping and variant calling for disease-associated variants [104,105], and thus further help to address the problem of missing heritability.

**Figure 3:**
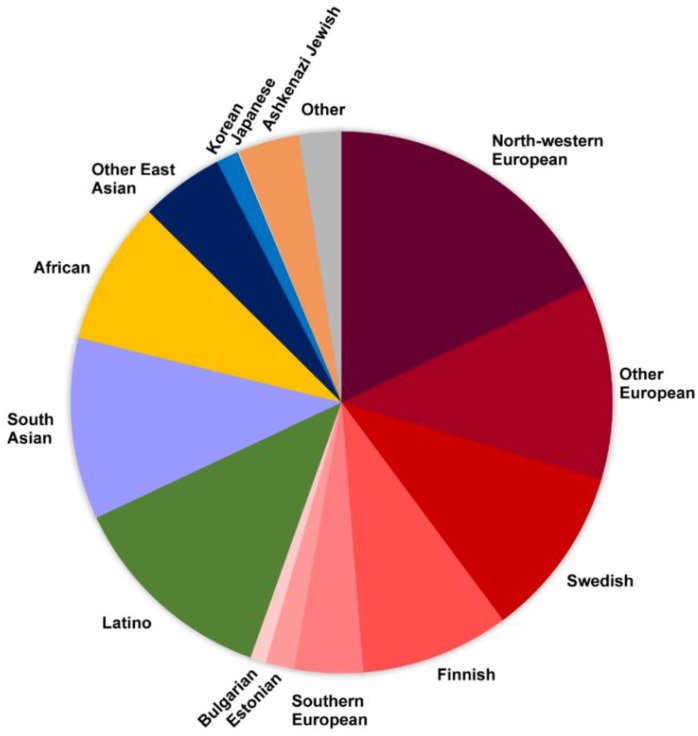
Populations represented in the gnomAD database. An example of various population exomes/genomes aggregated in the most comprehensive database, gnomAD (European populations are depicted in a spectrum of red colors).

Variome Resources: Another crucial component of the rare disease HTS bioinformatics pipelines is the assessment of the frequency of the variants identified in the patient by comparison against ‘untargeted populations’ or ‘normal variation’ databases. This step in variant interpretation can reduce the number of candidate variants several fold by deprioritizing the ones seen more frequently than expected in these databases, and thus focus analysis on the ultra/rare variants that are more likely to play a role. dbSNP [106], and databases such as Exome Aggregation Consortium (ExAC, 60,706 individual exomes) [37], DiscovEHR (50,726 individual exomes [38]), Genome Aggregation Database (gnomAD, 125,748 exomes and 15,708 genomes) [37] and TOPMed project BRAVO dataset (62,784 genomes) [107], aggregate exome/genome data on thousands of unrelated individuals not affected by severe pediatric genetic conditions, and thus represent invaluable resources. Even so, despite their large number of exomes/genomes, these databases are not representative of the global human population and variations, making interpretation difficult, especially in underrepresented populations (Figure 3). First, all of these resources use the GRCh37/hg19 and/or GRCh38/hg38 as the reference genome when calling the variants. Second, all of these resources predominantly contain the information on European ‘normal’ variation (e.g., 60% and 55% of ExAC and gnomAD data sets, respectively) (Figure 3), while other genomes are substantially under-represented (e.g., 67 Japanese individuals in gnomAD) or not at all (no information on Indigenous people) (Figure 3). This problem has been recognized and multiple efforts have been initiated to bridge these gaps, such as Iranome project [108], the Ashkenazi Jewish [109] reference panel, the Genome Russia project [110,111], as well as the Silent Genomes project (Canadian Indigenous people) [112]. Beyond these challenges with reference population data, another problem with the current population databases is that these aggregate predominantly SNVs. Thus, to effectively use WGS to uncover missing heritability, we will need both equitable representation of populations as well as robust methods to identify, compile and compare SVs across different populations.

Beyond the ‘normal variation’ resources, databases on variants already implicated in human disease are very important as well. These include already mentioned freely accessible database ClinVar [18], as well as Leiden Open Variation Database (LOVD) [20], Human Gene Mutation Database (HGMD) [113] and ClinGen resources [114]. Additional more specialized databases compile information on structural variants, such as a dbVar [115], a database housing over 3 million submitted structural variants (SSV) from 120 human studies or an HmtVar [116], a dataset of over 40,000 human mitochondrial variants.

Phenome Resources: Accurate and detailed phenotyping is essential for correct and timely gene/variant-disease associations. Beyond the resources on human genetic variations, the resources on human phenomes, such as OMIM [6] and ORPHANET [5] compile the information on human rare phenotypes, as well as information on corresponding genes in cases where the associations had been made. The Human Phenotype Ontology (HPO) database contains HPO terms, a standardized vocabulary used to describe/communicate phenotypic abnormalities associated with disorders [117]. The HPO vocabulary not only helps link genes to diseases but also helps in standardizing health records around the world and thus connecting patients with the same disease [118]. In terms of matchmaking tools, there are a number of resources that facilitated the matching of patients with similar rare phenotypes who may have the same candidate gene identified from exome/genome sequencing studies. These include GeneMatcher [119], PhenomeCentral [120], as well as Matchmaker Exchange [121]. Since thousands of genes remain to be associated with rare disease, these matchmaking tools are effectively helping the missing heritability problem (e.g., by providing additional evidence; more than one patient with the same novel genotype-phenotype association). Similarly, international efforts, like the International Rare Diseases Research Consortium (IRDiRC) [49], Canadian Organization for Rare Diseases (CORD), UK10k project [122], the National Institute of Health (NIH) initiatives, Undiagnosed Diseases Program [123] and others are determined to work together in order to resolve the missing heritability in RDs and to understand the genetic origin of disease [124].

## 4. Uncovering Missing Heritability—“No Longer Just Looking under the Lamppost”

In his William Allan Award address, Dr. Francis Collins used an “under the lamppost” search metaphor to illustrate his view of the difficulty associated with searching for genetic answers in the small regions of the genome only [124]. It relates to the story of a man losing his car keys in the street at night. He was only looking under the lamppost justifying that this is where he is likely to find his keys since this is where the light is. It is clear that in RDs, we are exhausting the “lamppost”, and thus it is time to search beyond for causes of “missing heritability”. With affordable sequencing of genomes, we are undeniably *en route* to find more variations (Table 1), to be inclusive of underrepresented populations (Figure 3), and well positioned to comb the genome base-by-base for answers. The search beyond the obvious truly opens windows to the wonders of genomics, and while it untangles some complexity, it informs us of another complexity of human genetic conditions that we did not even consider (e.g., complex mosaicisms [47], chromothripsis [23,24]).

In this review, we discuss the ‘missing heritability’ paradigm through the rare disease lens. Heritability (H^2^) H^2^ = $\frac{\mathrm{Var}\;G}{\mathrm{Var}\;P}=\;\frac{\mathrm{Var}\;G}{\mathrm{Var}\;G\;+\;\mathrm{Var}\;E}$ is a measure that estimates the proportion (0 to 1) of a phenotypic trait or phenotypic variance (Var P) that is genetic (Var G) in origin (i.e., it could not be explained by the environment (Var E) or random chance). We argue that missing heritability affects RDs in a fashion similar to common and complex diseases. Furthermore, we believe that given the fact that the majority of rare disease phenotypes are mostly due to genetics (Var G), RDs are the best phenotypic traits where causes of missing heritability, applicable also to common disease, can be effectively explored.

## Figures and Tables

**Table 1:** Examples of diagnoses facilitated by Whole Genome Sequencing (WGS).

Authors	Year	Gene	Disease	Type of Variation	Type of WGS	Ref.
**Kloosterman et al.**	2011	Multiple	Severe congenital abnormalities	De novo SV (chromothripsis)	SOLiD	[23]
**Gilissen et al.**	2014	*SHANK3*	Phelan-McDermid syndrome	De novo 66 kb deletion	Complete Genomics	[53]
**Gilissen et al.**	2014	*VPS13B*	Cohen syndrome	1.7 kb and 122 kb deletions	Complete Genomics	[53]
**Gilissen et al.**	2014	*MECP2*	Rett syndrome	De novo 0.6 kb deletion	Complete Genomics	[53]
**Gilissen et al.**	2014	*IQSEC2*	Intellectual disability	De novo 62 kb interspersed duplication	Complete Genomics	[53]
**Gilissen et al.**	2014	*SMC1A*	Cornelia de Lange syndrome	De novo 2.1 kb deletion	Complete Genomics	[53]
**Gilissen et al.**	2014	Multiple	16p11.2 deletion syndrome	De novo 611 kb deletion	Complete Genomics	[53]
**Gilissen et al.**	2014	*STAG1*	Intellectual Disability	De novo 382 kb deletion	Complete Genomics	[53]
**van Kuilenburg et al.**	2017	*DPYD*	DPDD	Large intragenic inversion	Illumina	[52]
**Chiu et al.**	2017	Multiple	Pulmonary alveolar proteinosis	425 kb deletion	Illumina	[63]
**Borràs et al.**	2017	*PKD1*	Polycystic kidney disease	Various, 18/19 probands	PacBio	[64]
**Cretu Stancu et al.**	2017	Multiple	Severe congenital abnormalities	De novo SV (chromothripsis)	ONT ^1^ + Illumina	[65]
**Alfares et al.**	2018	*PHOX2B*	Central hypoventilation syndrome	GCN (25) repeat expansion [+25]	Illumina	[54]
**Alfares et al.**	2018	*TPM3*	Nemaline myopathy 1	Large deletion	Illumina	[54]
**Alfares et al.**	2018	*TSC2*	Tuberous sclerosis type 2	De novo deep intronic SNV	Illumina	[54]
**Lionel et al. ^2^**	2018	*GPR143*	Ocular albinism	Deep intronic variant	Illumina	[55]
**Lionel et al. ^2^**	2018	*OTC*	Ornithine transcarbamylase deficiency	Deep intronic variant	Illumina	[55]
**Ostrander et al.**	2018	Multiple	Global developmental delay	Balanced inverted translocation	Illumina	[56]
**Ostrander et al.**	2018	*CDKL5*	Global developmental delay	De novo 63 kb tandem duplication	Illumina	[56]
**Tavares et al.**	2018	*BBS1*	Bardet-Biedl syndrome	Retrotransposon insertion	Illumina	[66]
**Cowley et al.**	2018	*SYNGAP1*	Epileptic encephalopathy	De novo 13 bp duplication	Illumina	[67]
**Miao et al.**	2018	*G6PC*	Glycogen storage disease type Ia	7.1 kb deletion	ONT ^1^	[68]
**Merker et al.**	2018	*PRKAR1A*	Carney complex	De novo 2184 bp deletion	PacBio	[69]
**Sanchis-Juan et al.**	2018	*ARID1B*	Coffin-Siris syndrome	De novo complex SV dupINVinvDEL	Illumina	[70]
**Sanchis-Juan et al.**	2018	*HNRNPU*	Seizures; Intellectual disability	De novo complex SV delINVdup	Illumina	[70]
**Sanchis-Juan et al.**	2018	*CEP78*	Cone-rod dystrophy; Hearing loss	complex homozygous SV delINVdel	Illumina	[70]
**Sanchis-Juan et al.**	2018	*CDKL5*	Birth asphyxia; Fetal distress	De novo complex SV dupINVdup	Illumina + ONT ^1^	[70]
**Ishiura et al.**	2018	*SAMD12*	BAFME ^3^	TTTCA and TTTTA repeat expansions	PacBio +ONT	[57]
**Ishiura et al.**	2018	*TNRC6A*	BAFME ^3^	TTTCA and TTTTA repeat expansions	PacBio + ONT	[57]
**Ishiura et al.**	2018	*RAPGEF2*	BAFME ^3^	TTTCA and TTTTA repeat expansions	PacBio + ONT	[57]
**Mizuguchi et al.**	2019	*SAMD12*	BAFME ^3^	4.6 kb intronic repeat insertion	PacBio	[71]

^1^ Oxford Nanopore Tech. ^2^ Lionel et al., reported 18 diagnoses by WGS; however, the majority was missed by exome panels since panels did not include the corresponding gene. The two deep intronic variants included in this table would not have been detected by exome sequencing approaches. ^3^ Benign adult familial myoclonic epilepsy.

**Table 2:** Examples of bioinformatics tools that facilitate comprehensive genome analyses.

Authors	Year	Tool	Method	Input ^1^	Variants Detected	Reference
**Abyzov et al.**	2011	CNVnator	Read Depth	PE^2^ Short read WGS	Copy Number Variants	[88]
**Rausch et al.**	2012	DELLY	Paired-ends, Read depth, Split-reads	Short read WGS	Structural Variants	[89]
**Calabrese et al.**	2014	MToolBox	Read re-alignment	WGS or WES	Mitochondrial Variants	[90]
**Layer et al.**	2014	LUMPY	Paired-ends, Read depth, Split-reads	PE short read WGS	Structural Variants	[91]
**Roller et al.**	2016	Canvas	Read Depth	WGS or WES	Copy Number Variations	[84,85]
**Chen et al.**	2016	Manta	Pair Read, Split Read	PE short read WGS	Indels, Structural Variants	[92]
**Dolzhenko et al.**	2017	Expansion-Hunter	Sequence-graph	PE short read WGS	Large Expansion of Short Tandem Repeats	[74]
**Ebler et al.**	2017	DIGTYPER	Breakpoint-Spanning, Split Alignments	PE short read WGS	Inversions, Tandem Duplications	[93]
**Liang et al.**	2017	Seeksv	Split Read, Discordant Paired-End, Read Depth, 2 Ends Unmapped	SE/PE ^2^ short read WGS	Structural Variants + Virus Integration	[94]
**Mousavi et al.**	2018	GangSTR	Enclosing, Fully Repetitive, Spanning and Off-target Fully Repetitive Read Pairs	PE short read WGS	Tandem Repeat expansions	[78]
**Kim et al.**	2018	Strelka2	Mixture-model	PE short read WGS	Single Nucleotide Variants, Indels	[95]
**Ye et al.**	2018	Pindel	Split-reads	PE short read WGS	Indels, Structural Variants (small and medium-size)	[96,97]
**Wala et al.**	2018	SvABA	Local assembly	PE short read WGS	Indels, Structural Variants (20–300 bp)	[98]
**Becker et al.**	2018	SVE/FusorSV	8 SV callers combination + Data mining	PE short read WGS	Deletions + Duplications + Inversions ^3^	[86]
**Antaki et al.**	2018	SV2	Supervised support vector machine classifiers	PE short read WGS	Deletions + Duplications	[87]

^1^ All tools take BAM files as input. MToolBoxaccepts FASTQ files. Strelka2, SV2, SvABA, ExpansionHunter, Manta also accept CRAM files, SV2 requires SVs to genotype, SNV VCF files and PED files. SVE/FusorSV accepts FASTQ, BAM and VCF files. SvABA also accepts SAM files. ^2^ PE = Paired-Ended; SE = Single-Ended ^3^ Other SVs could be explored if they are present in the training dataset.
